# Effect of volatile compounds produced by endophytic bacteria on virulence traits of grapevine crown gall pathogen, *Agrobacterium tumefaciens*

**DOI:** 10.1038/s41598-022-14864-w

**Published:** 2022-06-22

**Authors:** Faegheh Etminani, Behrouz Harighi, Ali Akbar Mozafari

**Affiliations:** 1 Department of Plant Protection, Faculty of Agriculture, University of Kurdistan, Sanandaj, Iran; 2 Department of Horticultural Science, Faculty of Agriculture, University of Kurdistan, Sanandaj, Iran

**Keywords:** Microbiology, Plant sciences

## Abstract

The volatile organic compounds (VOCs) produced by endophytic bacteria have a significant role in the control of phytopathogens. In this research, the VOCs produced by endophytic bacteria including *Serratia* sp. Ba10*, Pantoea* sp. Sa14*, Enterobacter* sp. Ou80*, Pseudomonas* sp. Ou22*, Pseudomonas* sp. Sn48 and *Pseudomonas* sp. Ba35, which were previously isolated from healthy domesticated and wild-growing grapevine were evaluated in terms of their effects on the virulence traits of *Agrobacterium tumefaciens* Gh1, the causal agent of crown gall disease. Based on the gas chromatography–mass spectrometry analysis, 16, 15, 14, 7, 16, and 15 VOCs have been identified with high quality in strains of Ba10, Sa14, Ou80, Ou22, Sn48, and Ba35, respectively. All endophytic bacteria produced VOCs that significantly reduced crown gall symptoms and inhibited the populations of *A. tumefaciens* Gh1 at different levels. Moreover, scanning electron microscopy analysis revealed various morphological abnormalities in the *A. tumefaciens* cells exposed to the VOCs produced by Ba35, Ou80, and Sn48 strains. The VOCs significantly reduced swarming-, swimming-, twitching motility and biofilm formation by *A. tumefaciens* Gh1. Our results revealed that VOCs could reduce the attachment of *A. tumefaciens* Gh1 cells to root tissues of grapevine cultivars Rashe and Bidane sefid, as well as chemotaxis motility towards root extract of both cultivars. Based on our results, it was shown that the antibacterial VOCs produced by endophytic bacteria investigated in the current study can manage crown gall disease and increase our knowledge on the role of VOCs in microbial interactions.

## Introduction

*Agrobacterium tumefaciens* is a Gram-negative, rod-shaped, non-spore-forming, motile bacterial pathogen causing crown gall disease worldwide^1^. The bacterial cells often enter via the wounds of roots, stems, and crowns of plants and are also able to transfer a fragment of the pathogenic plasmid (T-DNA) to the plant nuclear genome by a set of virulence (*vir*) genes located on the Ti plasmid^2^. Upon the infection of plants, *A. tumefaciens* provokes abnormal cell proliferation, resulting in tumor formation on woody plants such as pome fruit, stone fruit, and nut trees^3^. Motility including swimming and chemotaxis play important roles in the attachment, biofilm formation, and virulence traits of *A. tumefaciens* cells^4^.

Grapevine crown gall is known as one of the most economic diseases of grapevine worldwide^5^. Up to now, there are no effective chemical and physical control methods on grapevine crown gall disease^6^. In this regard, biological control seems to be a safe and cost-effective method for the management of soil-born plant pathogens^7^. Crown gall disease caused by *A. tumefaciens* on stone fruit trees has been successfully controlled using *Agrobacterium radiobacter* K84 and K1026 strains^8^. Several bacterial antagonists have been previously reported as biological control agents. In this regard, several studies have indicated that other bacteria, including nonpathogenic bacterium *Rahnella aquatilis* strain HX2, *Serratia plymuthica* IC1270, and *Pseudomonas fluorescens* Q8r1-96, were active against the growth of *A. tumefaciens* strains under in vitro conditions^9,10^.

Endophytic bacteria colonize the same ecological niche in plants as plant pathogens, and may have the potential to suppress the virulence of pathogenic microorganisms^11^. The increasing interest in endophytic bacteria for plant disease control is based on their ability to be biologically active against the pathogen through competition, antibiosis, and/or the induced resistance^12^. Bacteria have been reported to emit various volatile organic compounds (VOCs) with significant biological activities on a broad range of plant pathogens^13^. In general, volatile compounds are defined as small molecules (< 300 Da) with low boiling point and high vapor pressure. These molecules can easily spread between roots and microbes even in distances^14^. The chemical structures of bacterial volatiles are very diverse, so that in different groups like small aliphatic, aromatic molecules to large molecules can be observed^15^. Recently, the antibacterial activity has been reported in these volatile compounds^16,17^. It was also shown that VOCs released by *Bacillus amyloliquefaciens* SQR-9 and *Pseudomonas fluorescens* WR-1 can restrict the virulence traits of *Ralstonia solanacearum*^18,19^. In addition, previous studies indicated that VOCs released from *Bacillus* strains caused morphological abnormalities in *R. solanacearum* cells^20^.

In previous studies, we reported that some endophytic bacterial strains, which were isolated from the wild-growing and domesticated grapevine plants, had in vitro inhibition effects on *A. tumefaciens*, reduced gall formation *in planta*, and trigger defense response in grapevine plants against *A. tumefaciens*^21,22^. To the best of our knowledge, there is no research on the effect of VOCs produced by endophytic bacteria on *Agrobacterium tumefaciens*. Therefore, in the current study, we evaluate the effects of VOCs produced by these endophytic bacteria on *Agrobacterium tumefaciens*. The effects of VOCs on the growth rate; structural change; and virulence traits such as motility, chemotaxis, attachment, and biofilm formation were evaluated in the present study as well. Moreover, the major VOCs produced by endophytic bacteria against *Agrobacterium tumefaciens* were identified using gas chromatography-mass spectrophotometry (GC–MS).

## Results

### Antibacterial activity of VOCs produced by endophytic bacteria against *Agrobacterium tumefaciens*

According to the statistical analysis, significant differences were found between the treatments in terms of the colony size (F = 52.72; *P* < 0.0001) and the reduction of the populations (F = 65.33; *P* < 0.0001) of the *A. tumefaciens* Gh1 exposed to VOCs produced by endophytic bacteria compared to the control (Table 1).

**Table 1:** Analysis of variance (ANOVA) of biofilm production, swarming-, swimming-, twitching- motility, population, colony diameter and gall formation of *Agrobacterium tumefaciens* Gh1 under the effect of VOCs produced by endophytic bacteria.

Source of variation	df	Mean of Square						
		Biofilm	Swarming	Twitching	Swimming	population	Colony diameter	Gall weight
treatment	6	0.26*	19.25*	22.109*	19.20*	2.59*	27.93*	0.0003353*
Error	14	0.011	1.89	1.43	2.69	0.039	0.529	0.00002519
Cv (%)		16.03	13.49	12.42	14.23	13.12	6.76	15.87

*Significant at 1% probability level.

*df* degrees of freedom, *Cv* coefficient of variation.

**Figure 1:**
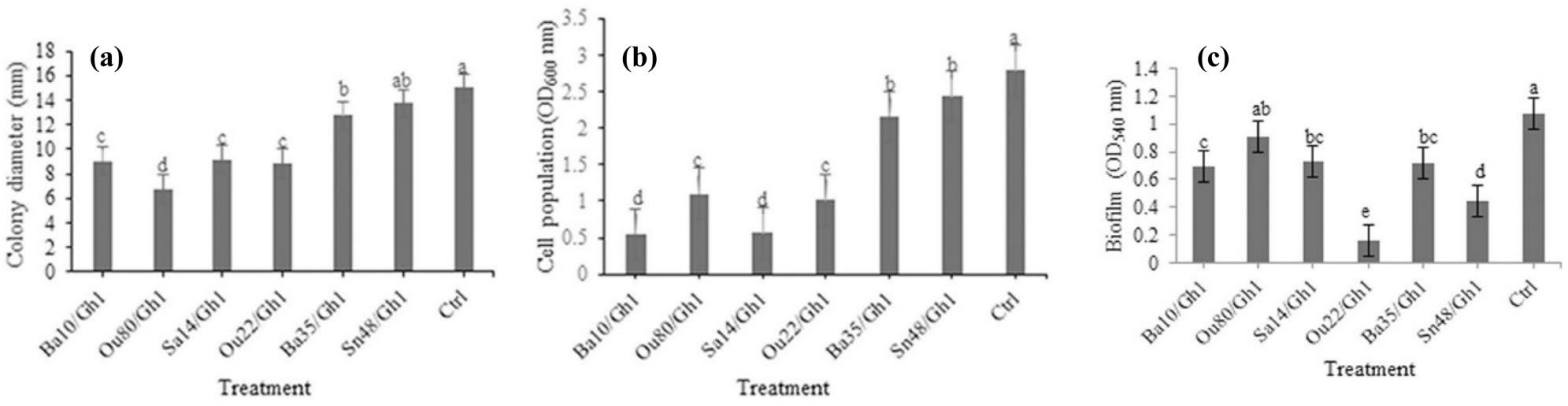
Effects of VOCs produced by *Serratia* sp. Ba10*, Enterobacter* sp. Enterobacter sp. Ou80*, Pantoea* sp. Sa14*, Pseudomonas* sp. Ou22, *Pseudomonas* sp. Ba35, and *Pseudomonas* sp. Sn48 on (**a**) colony diameter, (**b**) populations, and (**c**) biofilm formation of *Agrobacterium tumefaciens* Gh1 compared to the untreated control (Ctrl). Three replicates were used for each treatment. Error bars indicate SE of the three replicate. Different letters indicate significant differences (*P* = 0.05).

Most of the endophytic bacteria except *Pseudomonas* sp. Sn48 significantly reduced the colony growth diameter of the *A. tumefaciens* Gh1 at various levels compared to the control. Strain *Enterobacter* sp. Ou80 with about 55.14% reduction of the colony growth was found to have the highest effect, followed by *Pseudomonas* sp. Ou22, *Serratia* sp. Ba10, and *Pantoea* sp. Sa14 with 40.79%, 39.72%, and 38.58% reductions, respectively (Fig. 1a).

Moreover, our finding revealed that VOCs produced by endophytic bacteria reduced populations of *A. tumefaciens* Gh1. Additionally, the strains *Serratia* sp. Ba10, *Pantoea* sp. Sa14, and *Pseudomonas* sp. Ou22 decreased the populations of *A. tumefaciens* Gh1 to about 80.31%, 79.95%, and 63.84%, respectively (Fig. 1b).

### Effect of VOCs of endophytic bacteria on biofilm formation

As presented in Table 1, the result of ANOVA showed a significant difference among most of the treatments compared with the non-treated control (F = 22.81; *P* < 0.0001). The VOCs produced by endophytic bacteria revealed a significant inhibition effect on the biofilm formation by *A. tumefaciens* Gh1*.* The result indicates an 85.1%, 58%, and 35.6% decrease in the biofilm formation by *A. tumefaciens* Gh1 exposed to VOCs of *Pseudomonas* sp. Ou22, *Pseudomonas* sp. Sn48, and *Serratia* sp. Ba10 strains, respectively. As well, this is followed by the decrease in biofilm formation of about 31.9% after the treatment with *Pantoea* sp. Sa14 and *Pseudomonas* sp. Ba35 strains (Fig. 1c).

### Effect of VOCs on crown gall disease development

**Figure 2:**
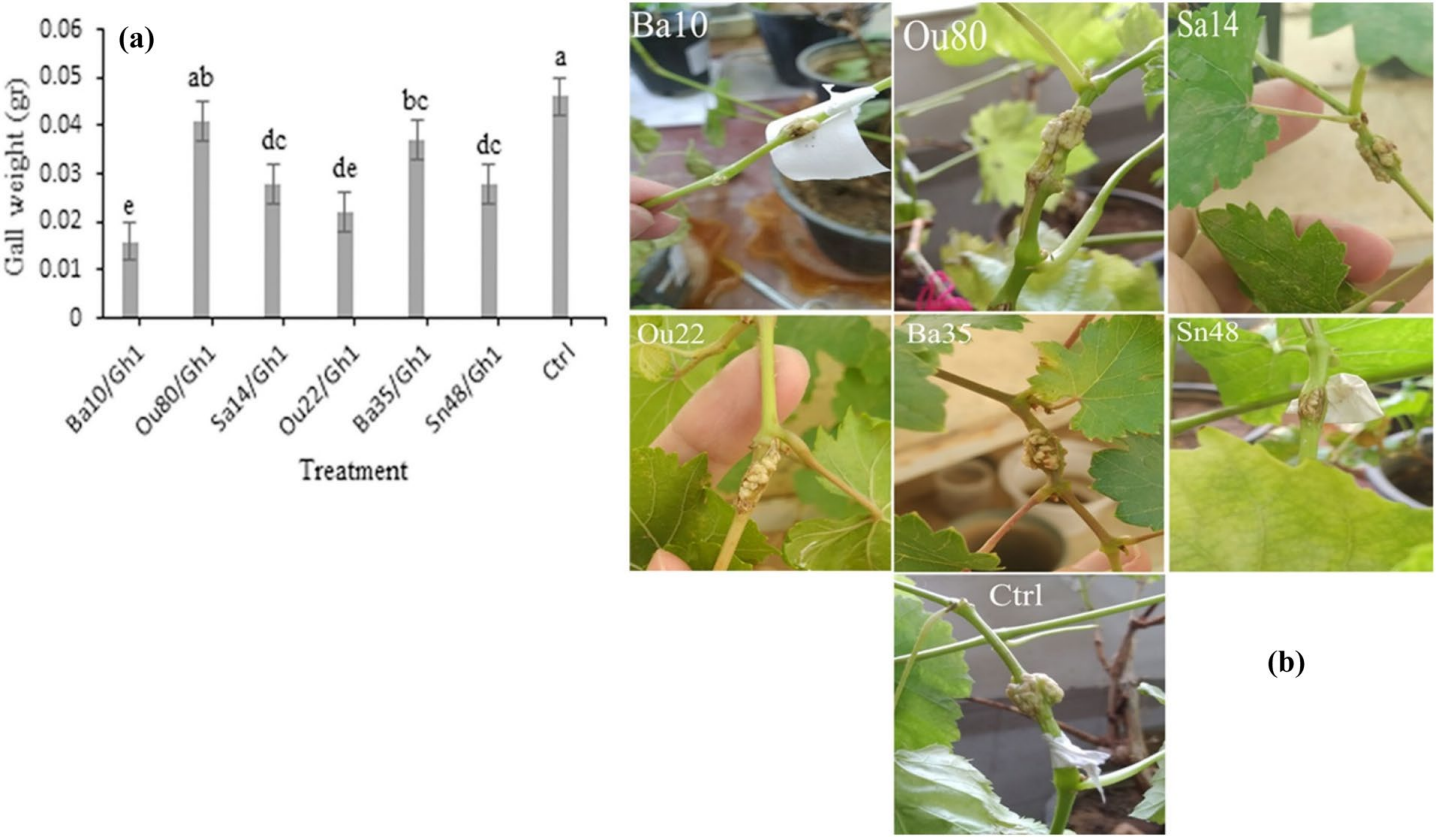
Comparison of fresh gall weight (**a**) and representative tumor (**b**) were shown on grapevine plantlets inoculated with *Agrobacterium tumefaciens* Gh1 (Ctrl) and *A. tumefaciens* cells exposed to VOCs produced by endophytic bacteria. Three replicates were used for each treatment. Error bars indicate SE of the three replicate. Different letters indicate significant differences (*P* = 0.05).

Statistical analysis revealed some significant differences between the treatments in terms of the inhibition of crown gall development (F = 13.31; *P* < 0.0001) by *A. tumefaciens* Gh1 exposed to VOCs produced by endophytic bacteria compared to the control (Table 1). The obtained results reveal that VOCs produced by the strains *Serratia* sp. Ba10 and *Pseudomonas* sp. Ou22 with about 65%, 52%, respectively had the highest decreasing effects on gall weight production by *A. tumefaciens* Gh1 followed by *Pantoea* sp. Sa14 and *Pseudomonas* sp. Sn48 with about 40% decreasing effects (Fig. 2a and b).

### Effect of VOCs on cell morphology

**Figure 3:**
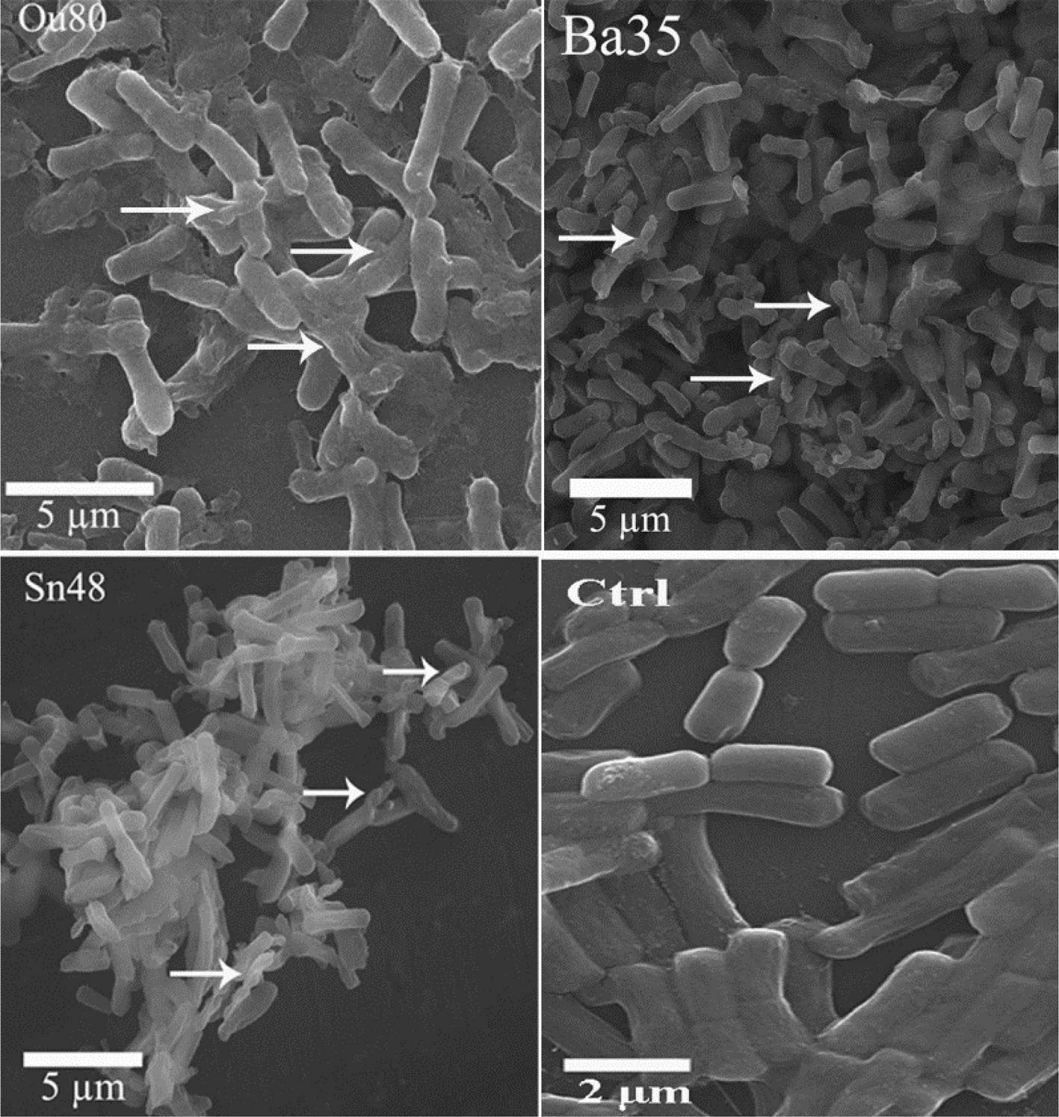
Scanning electron microscopic analysis of the cellular morphology of *A. tumefaciens* exposed by the VOCs produced by *Pseudomonas* sp. Ba35, *Enterobacter* sp. Ou80, and *Pseudomonas* sp. Sn48. The cells showed normal short rod in the untreated control (Ctrl). Many of the cells having rough, wrinkled surfaces, and cracks after exposure to the VOCs of endophytic bacteria. The white arrows indicated the damaged cells.

SEM analysis revealed various morphological abnormalities in the *A. tumefaciens* cells exposed to the VOCs produced by *Pseudomonas* sp. Ba35, *Enterobacter* sp. Ou80, and *Pseudomonas* sp. Sn48 strains compared to the non-treated control (Fig. 3). The cells of the non-treated control showed normal growth, whereas more than 30% of the *A. tumefaciens* cells showed rough, wrinkled surfaces and cracks following co-cultivation with endophytic bacteria.

### Effect of VOCs on the motility behaviors of *A. tumefaciens*

**Figure 4:**
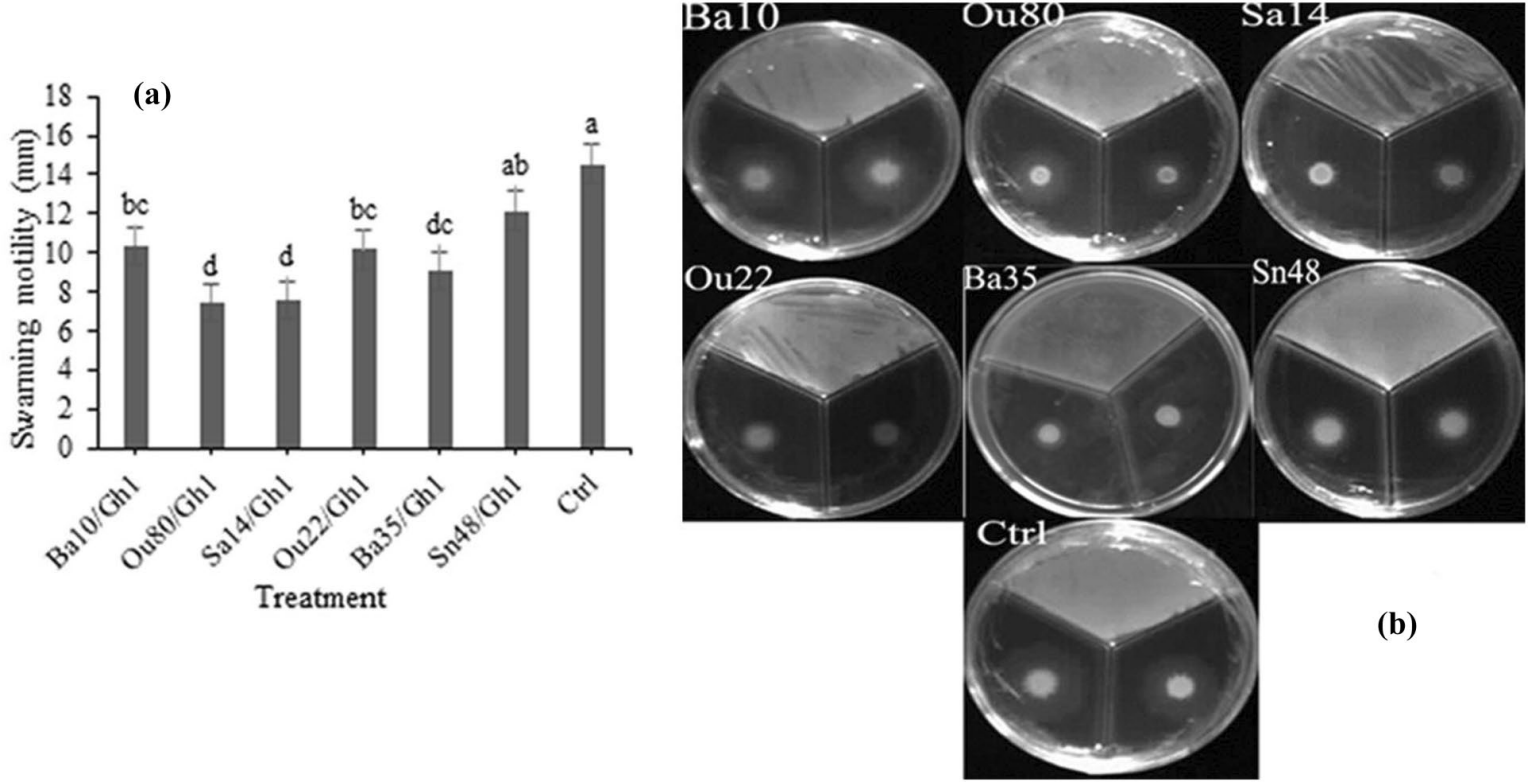
Effects of VOCs produced by *Serratia* sp. Ba10*, Enterobacter* sp. *Enterobacter* sp. Ou80*, Pantoea* sp. Sa14*, Pseudomonas* sp. Ou22, *Pseudomonas* sp. Ba35, and *Pseudomonas* sp. Sn48 on swarming motility of *Agrobacterium tumefaciens* Gh1 compared to the untreated control (Ctrl). The diameter of motility zone (**a**), and representative plate of swarming motility assay (**b**) were shown. Two replicates were used for each treatment. The experiment was repeated three times. Error bars indicate SE of the three replicate. Different letters indicate significant differences (*P* = 0.05).

According to statistical analysis in this study, significant differences were existed between the treatments in the swarming (F = 10.16; *P* = 0.0002), swimming (F = 7.12; *P* = 0.0012), and twitching (F = 15.45; *P* < 0.0001) assessments (Table 1). Our finding revealed that swarming motility of *A. tumefaciens* Gh1 significantly inhibited after exposure to VOCs of endophytic bacterial strains for 72 h. As illustrated in Figs. 4a &b, the strains *Enterobacter* sp. Ou80 and *Pantoea* sp. Sa14*,* with a mean of 7.5 mm showed the highest inhibition effects of *A. tumefaciens* Gh1 cells followed by *Pseudomonas* sp. Ba35, *Pseudomonas* sp. Ou22 and *Serratia* sp. Ba10 with the means of 9.1, 10.1, and 10.4 mm, respectively, as compared to the control with a mean of 14.6 mm.

**Figure 5:**
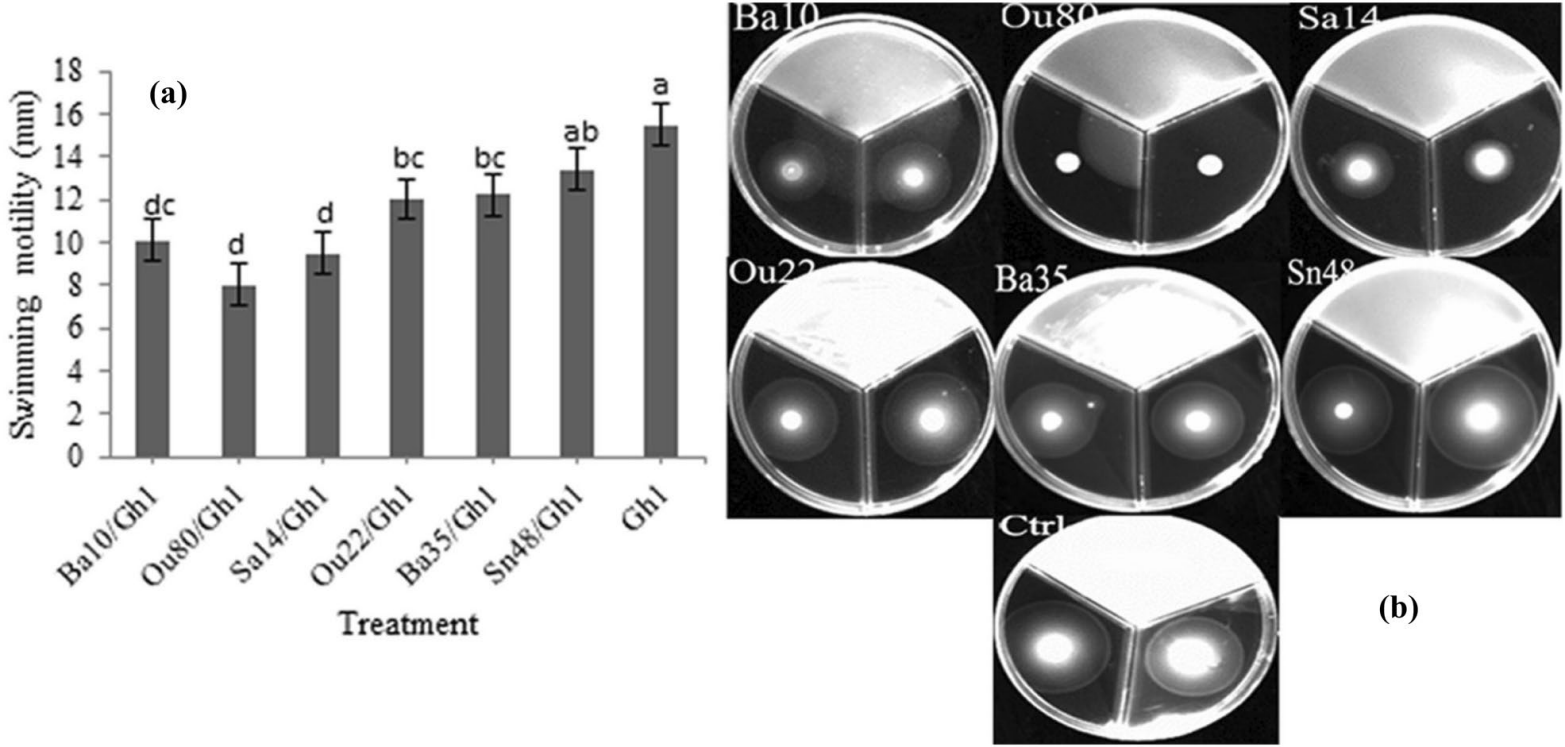
Effects of VOCs produced by *Serratia* sp. Ba10*, Enterobacter* sp. Ou80*, Pantoea* sp. Sa14*, Pseudomonas* sp. Ou22, *Pseudomonas* sp. Ba35, and *Pseudomonas* sp. Sn48 on swimming motility of *Agrobacterium tumefaciens* Gh1 compared to the untreated control (Ctrl). The diameter of motility zone (**a**), and representative plate of swimming motility assay (**b**) were shown. Two replicates were used for each treatment. The experiment was repeated three times. Error bars indicate SE of the three replicate. Different letters indicate significant differences (*P* = 0.05).

**Figure 6:**
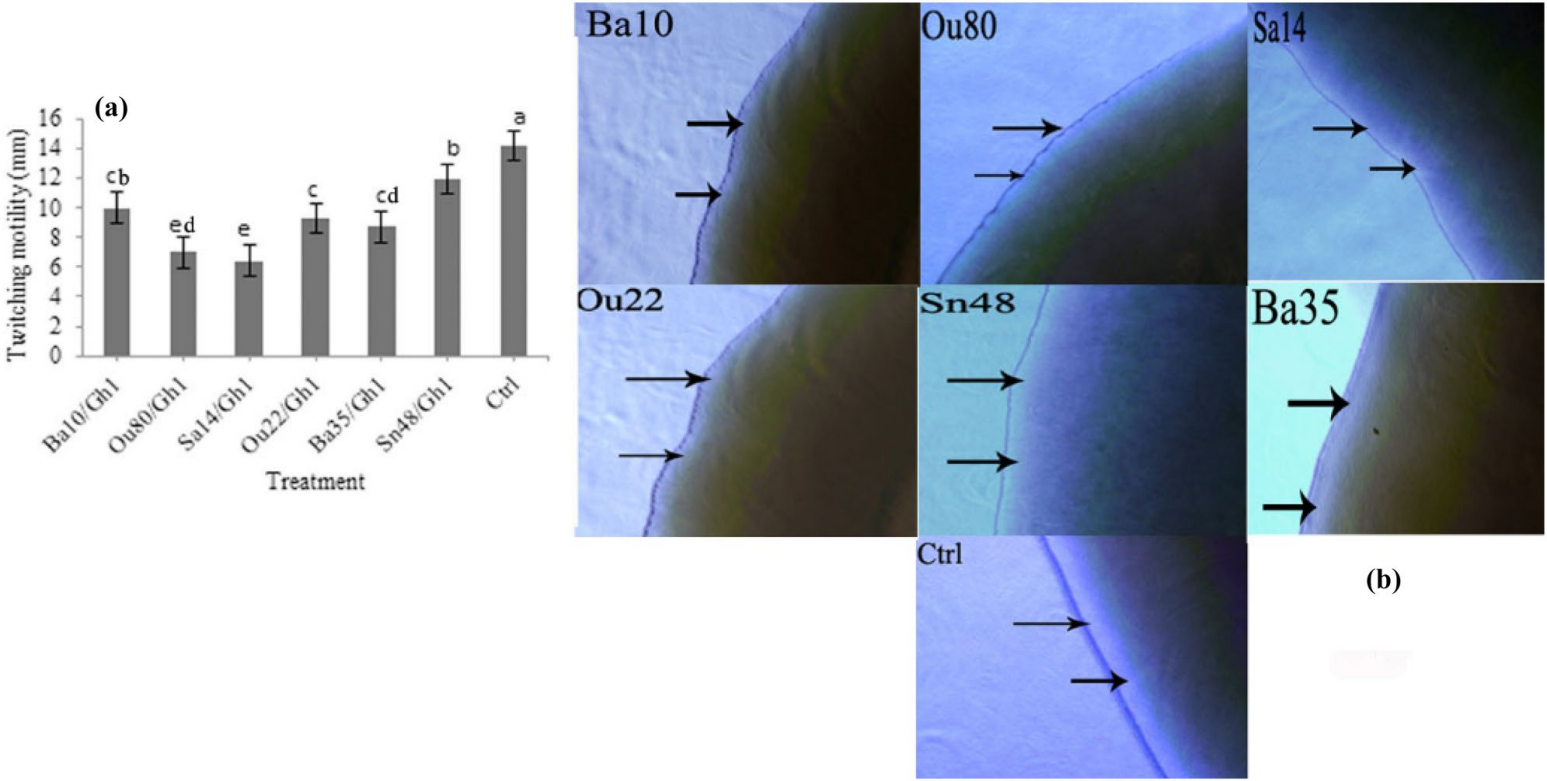
Effects of VOCs produced by *Serratia* sp. Ba10*, Enterobacter* sp. Ou80*, Pantoea* sp. Sa14*, Pseudomonas* sp. Ou22, *Pseudomonas* sp. Ba35, and *Pseudomonas* sp. Sn48 on twitching motility of *Agrobacterium tumefaciens* Gh1. (**a**) colony diameter, and (**b**) arrows indicate the wider peripheral colony edges of *A. tumefaciens* Gh1 cells in control (Ctrl) compared to the cells exposed to VOCs. Three replicates were used for each treatment. Error bars indicate SE of the three replicate. Different letters indicate significant differences (*P* = 0.05).

The VOCs produced by *Enterobacter* sp. Ou80, *Pantoea* sp. Sa14 and *Serratia* sp. Ba10 inhibited swimming motility of *A. tumefaciens* Gh1 to 8.0, 9.5, and 10.1 mm, respectively as compared to control with 15.5 mm. Correspondingly, these had the highest negative effect after 48 h (Fig. 5a and b). Similarly, twitching motility significantly reduced to 6.4 mm by *Pantoea* sp. Sa14 and 6.9 mm by *Enterobacter* sp. Ou80 following 8.7, 9.3, and 9.9 mm by *Pseudomonas* sp. Ba35, *Pseudomonas* sp. Ou22, and *Serratia* sp. Ba10, respectively compared to control with 14.2 mm (Fig. 6a). Microscopic examination of the twitching motility exhibited that the circumferential colony edge of *A. tumefaciens* Gh1 was significantly wider in the non-exposed control compared to those exposed with the *Serratia* sp. Ba10, *Enterobacter* sp. Ou80*,* P*antoea* sp. Sa14*,* and *Pseudomonas* sp. Ou22 volatiles (Fig. 6b).

### Effect of VOCs on the chemotaxis behavior of *A. tumefaciens*

**Figure 7:**
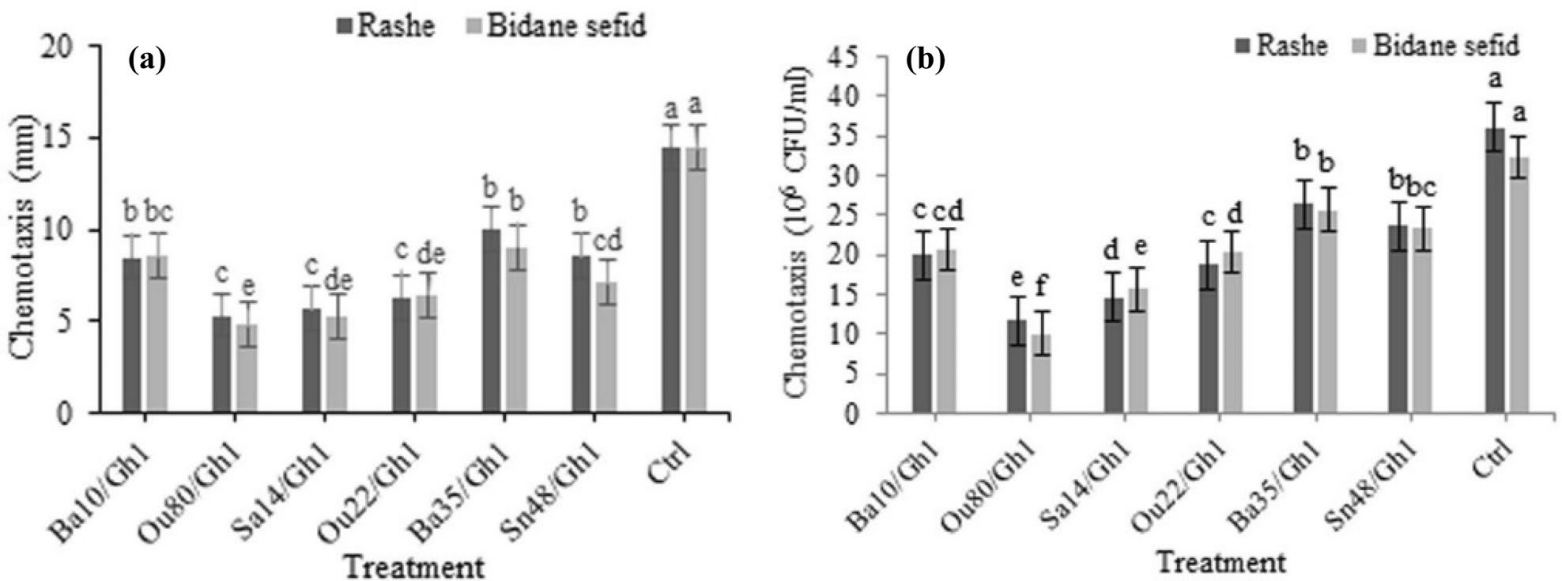
Effects of VOCs produced by *Serratia* sp. Ba10*, Enterobacter* sp. Ou80*, Pantoea* sp. Sa14*, Pseudomonas* sp. Ou22, *Pseudomonas* sp. Ba35, and *Pseudomonas* sp. Sn48 on chemotaxis behavior of *Agrobacterium tumefaciens* Gh1 toward root extract of grapevine cultivars Rashe, and Bidane sefid, (**a**) colony diameter, and (**b**) number of cells migrated towards the attractant compared to the untreated control (Ctrl). Three replicates were used for each treatment. Error bars indicate SE of the three replicate. Different letters indicate significant differences (*P* = 0.05).

Based on the results of ANOVA analysis, significant differences were found among all the treatments in the chemotaxis assays both in terms of the colony diameter (F = 23.01; *P* < 0.0001) and the number of cells migrated towards attractant (F = 56.86; *P* < 0.0001) (Table 2). Based on the results obtained from chemotaxis assay, *A. tumefaciens* Gh1 cells treated by VOCs of endophytic bacteria showed a significantly lower chemotaxis motility towards the hole containing *vitis vinifera* (Rashe and Bidane sefid cultivar) root extract compared to the control (Fig. 7a). Moreover, VOCs produced by *Enterobacter* sp. Ou80, *Pantoea* sp. Sa14 and *Pseudomonas* sp. Ou22 strains showed the highest inhibition effects of chemotaxis of *A. tumefaciens* toward Rashe cultivar compared with control by 63.21%, 61%, and 56.3% respectively.

**Table 2:** Analysis of variance (ANOVA) of chemotaxis and attachment of *Agrobacterium tumefaciens* Gh1 towards the root exudate and the root tissue of grapevine cultivars Rashe and Bidane sefid under the effect of VOCs produced by endophytic bacteria.

Source of variation	df	Means of Square			
		Chemotaxis (colony diameter)	Chemotaxis (× 10^6^ CFU)	Attachment (un-wounded root)	Attachment (wounded root)
cultivar	1	2.27	1.92	1.037	0.438
bacteria	6	61.84**	343.80**	18.25**	15.564**
Cultivar*bacteria	6	0.50	4.92	0.129	0.221
Error	28	1.25	2.833	0.34	0.306
Cv (%)		13.70	7.8	14.59	11.86

*df* degrees of freedom, *Cv* coefficient of variation.

**Significant at 1% probability level.

Similarly, VOCs produced by strains *Enterobacter* sp. Ou80, *Pantoea* sp. Sa14, *Pseudomonas* sp. Ou22 and *Pseudomonas* sp. Sn48 reduced chemotaxis of *A. tumefaciens* Gh1 toward Bidane sefid cultivar extraction by 76.9%, 63.4%, 55.5%, and 50.8% compared to the control, respectively. As shown in Fig. 7b, the VOCs produced by *Enterobacter* sp. Ou80, *Pantoea* sp. Sa14, *Pseudomonas* sp. Ou22, and *Serratia* sp. Ba10 strains reduced the motility of *A. tumefaciens* Gh1 cells toward Rashe cultivar compared with control by 67.61%, 59.27%, 48.16%, and 44.44% respectively. Similarly, VOCs produced by strains *Enterobacter* sp. Ou80 and *Pantoea* sp. Sa14, inhibited the motility of *A. tumefaciens* Gh1 cells toward Bidane sefid cultivar extraction compared with control by 69.06%, and 51.56%, respectively. No significant difference was found in chemotaxis of *A. tumefaciens* Gh1 cells treated by endophytic bacterial strains toward root extract of both Rashe and Bidane sefid cultivars (Table 2).

### Effect of VOCs of endophytic bacteria on *A. tumefaciens* root attachment

The ability of *Agrobacterium tumefaciens* to attach to root segments after the treatment with strains *Serratia* sp. Ba10, *Pantoea* sp. Sa14, *Enterobacter* sp. Ou80, *Pseudomonas* sp. Ou22, *Pseudomonas* sp. Ba35 and *Pseudomonas* sp. Sn48 was investigated in both wounded and unwounded roots of Rashe and Bidane sefid cultivars. Based on the results of ANOVA, significant differences existed among all the treatments in terms of the attachment of treated-*A. tumefaciens* Gh1 cells to unwounded (F = 24.73; *P* < 0.0001) and wounded roots (F = 23.90; *P* < 0.0001).

**Figure 8:**
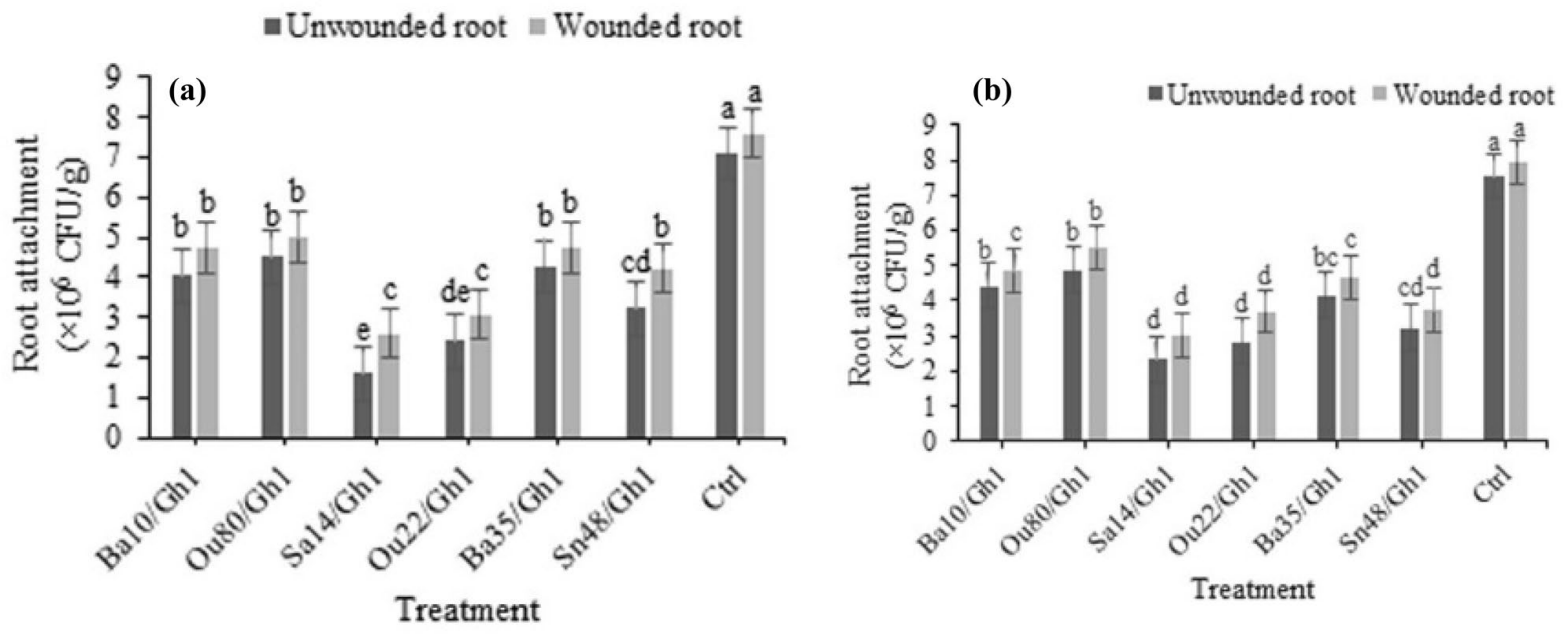
Effect of VOCs produced by *Serratia* sp. Ba10*, Enterobacter* sp. Ou80*, Pantoea* sp. Sa14*, Pseudomonas* sp. Ou22, *Pseudomonas* sp. Ba35, and *Pseudomonas* sp. Sn48 on grapevine root attachment (**a**) Rashe, and (**b**) Bidane Sefid cultivars by *Agrobacterium tumefaciens* Gh1 compared to the untreated control (Ctrl). Error bars indicate standard error of three replicates and different letters describe significant differences at *P* = 0.05 within the same data group.

In the wounded roots of Rashe cultivar, the highest inhibition belonged to *Pantoea* sp. Sa14 with a 65.78% reduction, followed by *Pseudomonas* sp. Ou22 (59.74%), *Pseudomonas* sp. Sn48 (44.8%), *Serratia* sp. Ba10 and *Pseudomonas* sp. Ba35 (37.76%), and *Enterobacter* sp. Ou80 (34.21%), respectively. Furthermore, our finding showed a reduction in the attachment of the exposed *A. tumefaciens* Gh1 cells to unwounded roots by *Pantoea* sp. Sa14 (77.46%), *Pseudomonas* sp. Ou22 (66.20%), *Pseudomonas* sp. Sn48 (54.6%), *Serratia* sp. Ba10 (43.23%) and *Enterobacter* sp. Ou80 (36.62%) compared to the control (Fig. 8a).

VOCs produced by the strains *Pantoea* sp. Sa14*, Pseudomonas* sp. Ou22, *Pseudomonas* sp. Sn48, and *Pseudomonas* sp. Ba35 with 61.96% 53.52%, 53.27, and 41.30% reduction, respectively showed greater effects on the attachment of *A. tumefaciens* Gh1 cells to the wounded roots of Bidane sefid, compared to the control. Additionally, in unwounded roots, VOCs produced by strains *Pantoea* sp. Sa14, *Pseudomonas* sp. Ou22, and *Pseudomonas* sp. Sn48 with 69.09%, 62.59%, and 57.42% reduction, respectively, exhibited greater effects on the attachment of *A. tumefaciens* Gh1 cells compared to the control (Fig. 8b).

### Identification of VOCs produced by endophytic bacteria

VOCs produced by endophytic bacteria were analyzed by GC–MS and compared with the volatiles retrieved from the control. Based on the GC–MS analysis 16, 15, 14, 7, 16, and 15 VOCs have been identified with high quality in the strains of *Serratia* sp. Ba10, *Pantoea* sp. Sa14, *Enterobacter* sp. Ou80, *Pseudomonas* sp. Ou22, *Pseudomonas* sp. Sn48, and *Pseudomonas* sp. Ba35, respectively in interaction with *A. tumefaciens* Gh1 (chromatographs for each strain are available as individual in the supplementary file 1). The VOCs dodecane, eicosane, hexadecane, and tetradecane were produced by all the tested bacterial strains. Among the tested strains, only *Serratia* sp. Ba10 was able to produce the VOC methyl stearate. The strain *Pseudomonas* sp. Sn48 specifically produced Bicyclo[4.4.0]dec-1-ene, 2-isopropyl-5-methyl-9-methylene, ethylbenzene, and styrene volatiles, while only the strain *Pseudomonas* sp. Ba35 produced (1S,4S,4aS)-1-Isopropyl-4,7-dimethyl-1,2,3,4,4a,5-hexahydronaphthalene, linalool, and 1-alpha-terpineol as VOCs. The main VOC produced by *Pseudomonas* sp. Ou22 were dodecane, tetradecane, and benzen, 1,3-dimethyl with a peak areas of 15.14% (RT = 12.56), 12.93% (RT = 17.85), and 10.81% (RT = 3.91), respectively, in the high quality. The main VOC produced by *Pantoea* sp. Sa14, *Enterobacter* sp. Ou80, and *Serratia* sp. Ba10 was 9-Octadecenoic acid, methyl ester with peak areas of 32.99% (RT = 16.67), 17.93% (RT = 16.48), and 40.42% (RT = 16.67), respectively (Table 3).

**Table 3:** Volatile organic compounds produced by *Serratia* sp. Ba10, *Pantoea* sp. Sa14, *Enterobacter* sp. Ou80, *Pseudomonas* sp. Ou22, *Pseudomonas* sp. Sn48, and *Pseudomonas* sp. Ba35 strains against *Agrobacterium tumefaciens* Gh1 and detected by GC–MS analysis.

Volatile organic compounds	*Serratia* sp. Ba10		*Enterobacter* sp. Ou80		*Pantoea* sp. Sa14		*Pseudomonas* sp. Ou22		*Pseudomonas* sp. Ba35		*Pseudomonas* sp. Sn48	
	RT (min)	RPA (%)	RT (min)	RPA (%)	RT (min)	RPA (%)	RT (min)	RPA (%)	RT (min)	RPA (%)	RT (min)	RPA (%)
(1S,4S,4aS)-1-Isopropyl-4,7-dimethyl-1,2,3,4,4a,5-hexahydronaphthalene	–	–	–	–	–	–	–	–	15.85	5.86	–	–
2, 4-Di-tert-butylphenol	10.55	4.19	10.55	5.47	10.55	5.84	–	–	14.23	5.73	14.24	4.80
-9,12-Octadecadienoic acid, methyl ester	16.62	11.52	16.42	3.65	16.61	8.56	–	–	–	–	–	–
-9-Octadecenoic acid, methyl ester	16.67	40.42	16.48	17.93	16.67	32.99	–	–	–	–	–	–
Hexadecanoic acid, methyl ester	15.01	4.66	14.64	3.40	15.00	4.29	–	–	–	–	–	–
Benzene, 1,2,3-trimethyl-	4.06	2.23	3.37	1.55	3.68	5.73	–	–	6.69	4.25	6.71	3.44
Benzene, 1-ethyl-3-methyl-	3.29	2.77	–	–	3.29	5.60	5.98	9.08	6.26	1.60	6.16	4.38
Benzene, 1,2,4-trimethyl	4.05	0.84	–	–	–	–	–	–	–	–	6.28	1.18
Benzene, 1,3-dimethyl-	–	–	–	–	–	–	3.91	10.81	4.58	6.46	4.60	5.94
Benzene, 1-ethyl-2-methyl-	3.51	0.84	3.29	6.59	3.51	1.50	–	–	6.14	3.84	–	–
Bicyclo[4.4.0]dec-1-ene, 2-isopropyl-5-methyl-9-methylene-	–	–	–	–	–	–	–	–	–	–	15.87	3.29
Decane	3.73	0.99	3.73	2.10	3.73	1.89	–	–	6.75	3.72	6.77	2.89
Dodecane	6.44	3.00	6.44	4.84	6.44	3.87	12.56	15.14	9.94	6.82	9.95	5.94
Eicosane	12.65	4.33	10.30	4.77	13.72	8.04	22.63	9.63	19.44	4.85	18.74	5.26
Octadecane	13.72	3.55	13.14	6.46	19.22	1.85	–	–	17.42	4.48	17.44	3.48
Heptadecane	–	–	11.81	3.35	12.65	1.47	–	–	–	–	16.36	0.59
Hexadecane	11.53	3.12	11.53	4.34	11.53	4.53	20.19	4.69	15.2	7.25	15.22	5.62
Ethylbenzene	–	–	–	–	–	–	–	–	–	–	4.47	0.72
L-.alpha.-Terpineol	–	–	–	–	–	–	–	–	9.90	2.34	–	–
Linalool	–	–	–	–	–	–	–	–	8.43	1.53	–	–
Mesitylene	3.37	2.97	3.68	5.44	3.38	1.79	–	–	–	–	–	–
Methyl stearate	16.91	1.35	–	–	–	–	–	–	–	–	–	–
o-Xylene	–	–	–	–	–	–	4.38	5.75	4.97	7.22	4.99	5.43
Styrene	–	–	–	–	–	–	–	–	–	–	4.96	0.93
Tetradecane	9.11	3.58	9.11	4.54	9.11	4.95	17.85	12.93	12.73	8.16	6.44	9.36

Chromatographs for each strain are available as individual in the supplementary material.

*RT* retention time, *RPA* relative peak area, – VOCs not detected.

## Discussion

Biological control of plant pathogens using beneficial bacteria can be considered as a safe and efficient method for reducing disease incidence. Biocontrol agents act through various mechanisms, and VOCs have gained increasing interest due to participating in the cross-talk between microbes and other organisms in the environment^23^. Numerous reports of VOCs produced by bacteria have previously shown the inhibition effects of bacterial plant pathogens. The VOC dimethyl disulfide produced by two rhizospheric bacteria, including *Pseudomonas fluorescens* and *Serratia plymuthica*, with antibacterial effects on two plant bacterial pathogens *Agrobacterium tumefaciens* and *Agrobacterium vitis* have been previously reported^10^. *Pseudomonas fluorescens* WR-1 emit volatile compounds such as benzothiazole and 1-methyl naphthalene against the tomato pathogen *Ralstonia solanacearum* with a bacteriostatic effect^18^. Moreover, *Bacillus amyloliquefaciens* FZB42, *Bacillus artrophaeus* LSSC22, and *Bacillus amyloliquefaciens* SQR-9 volatiles significantly inhibited the physiology, morphology, and virulence factors of *Ralstonia solanacearum,* there by resulting in decreased wilt disease^19,24^. In another study, the VOCs produced by *Bacillus subtilis* FA26, which could adversely affect the ultra-structure of cells of *Clavibacter michiganenesis* ssp. *sepedonicus,* as the causal agent of bacterial ring rot of potato have been reported^16^. In addition, some recent studies demonstrated that volatiles emitted from *Bacillus* strain D13 could reduce the cell motility of *Xanthomonas oryzae* pv. *oryzae*^17^. Previous report indicated that sesquiterpene albaflavenone, as a VOC compound produced by *Streptomyces albidoflavus,* has an antibacterial activity against *Bacillus subtilis*^25^*.*

Endophytic bacteria spend their life within the plant tissues without leading to development of any disease, and they produce a wide range of volatile organic compounds with an antimicrobial activity^26^. In previous studies, endophytic bacteria were isolated from healthy domesticated and wild-growing grapevine in Iran, and some strains were detected with antagonistic effects on *Agrobacterium tumefaciens*, which is the causal agent of crown gall disease under both in vitro and in vivo conditions^21^. In the present study, these strains were screened for their antagonistic activity against *A. tumefaciens* via the production of volatile organic compounds. *A. tumefaciens* cells require chemotaxis, motility, and the attachment to virulence^4^. Our finding showed that VOCs produced by endophytic bacteria could significantly inhibit the growth of *A. tumefaciens* and crown gall symptoms. Our results reveal various inhibition effects on the chemotaxis, motility, biofilm formation, and root attachment of *A. tumefaciens* following the exposure to VOCs of endophytic bacteria.

Previous studies have also revealed that swimming is the most common motility behavior of *A. tumefaciens* and there is no evidence related to both swarming and twitching motility^27^. In contrast, our results demonstrate that *A. tumefaciens* cells have three forms of motility including swarming, swimming, and twitching. *A. tuemfaciens* senses and chemotaxis towards the plant exudates^28,29^. The results presented in the current study reveal that VOCs produced by endophytic bacterial strains could significantly inhibit all three forms of motility and chemotaxis. This phenomenon was further confirmed in the gall formation *in planta* and root attachment assay. As well, this finding is in agreement with previous studies showing that active motility and chemotaxis are required for *A. tumefaciens* attachment^4,27,30^. The VOCs produced by endophytic bacteria reduced the populations of *A. tumefaciens* Gh1. Accordingly, these results show that VOCs might keep *A. tumefaciens* cells away from the plants not only by inhibiting its movement and the subsequent attachment, but also by reducing its populations.

It was shown that *A. tumefaciens* can form biofilm on abiotic and plant surfaces, as well as participating in plant tissues attachment^31^. Nonmotile mutants were significantly deficient in biofilm formation under static conditions. Under flowing conditions, however, the aflagellate mutant rapidly formed aberrantly dense, tall biofilms^27^. Our results indicate no direct relationship exists between reduction of motility and biofilm formation and the root attachment of *A. tumefaciens* cells exposed to VOCs emitted by individual endophytic bacterial strains. In the present research, the attachment of *A. tumefaciens* cells to grapevine Rashe, and Bidane Sefid cultivars was tested both in wounded and unwounded roots. Correspondingly, the obtained results indicate that *A. tumefaciens* attached to the grape root of both cultivars at a high population. Moreover, no significant differences were observed between the attachment to wounded and unwounded grapevine roots. This result is in agreement with the previously reported equal attachment of *A. tumefaciens* bv.1 to both wounded and unwounded grape roots^30^.

Electron microscopic analysis of the non-treated *A. tumefaciens* cells indicated normal growth, while the cells were damaged in the presence of VOCs of *Enterobacter* sp. Ou80, *Pseudomonas* sp. Ba35, and *Pseudomonas* sp. Sn48. Accordingly, this result is consistent with previous studies in which the abnormality of the pathogenic cells was observed after the exposure to bacterial VOCs^16,17,20^. Our previous study revealed that defense-related genes such as *PR1, PR2,* and *PR4,* were upregulated in plants treated with the strain *Pseudomonas* sp. Sn48^22^. This result suggests that VOCs produced by this strain could not only inhibit growth and motility traits of *A. tumefaciens,* but it could also induce a systemic resistance.

The GC–MS analysis showed some differences in VOCs profiles among endophytic bacterial strains. The VOCs dodecane, tetradecane, hexadecane, and eicosane were produced by all the strains tested. There have been several reports on the antibacterial and antifungal activities of these compounds^32–34^. The exposure to these VOCs decrease the bacterial ability to form biofilm and also bring negative effects on motility^35^. Under our experimental condition, the main VOC produced by *Serratia* sp. Ba10, *Pantoea* sp. Sa14, and *Enterobacter* sp. Ou80 strains was 9-Octadecenoic acid, methyl ester. Correspondingly, this fatty acid has been reported with both biosurfactant and anti-biofilm activity, so it could inhibit bacterial motility^36^. Notably, biosurfactants can reduce surface tension properties such as biofilm formation and attachment. It is suggested that the negative effects of these strains on motility and attachment of *A. tumefaciens* cells at least in part, are related to the production of 9-Octadecenoic acid and other fatty acids, including hexadecanoic acid methyl ester. Furthermore, these compounds have been widely described with antibacterial and antifungal activities in various studies^37,38^.

*Pseudomonas* sp. Ou22, *Pseudomonas* sp. Ba35, and *Pseudomonas* sp. Sn48 strains belonging to the *Pseudomonas* genus, produce various VOCs. Of which, the most abundant volatiles detected were long-chain alkenes such as dodecane, tetradecane, hexadecane, and aromatic hydrocarbon o-xylene, and Benzene, 1,3-dimethyl. In addition, *Pseudomonas* species, which are frequently reported as endophytic bacteria are well-known as plant growth-promoting bacteria by causing inhibition effects on plant pathogens^39^. Previous studies reported dodecane, tetradecane, and other VOCs released by *Pseudomonas* spp. with growth-promoting effect in *Vigna radiate* seedlings^40^. They find new insight on plant beneficial effects of VOCs produced by *Pseudomonas* spp. Our results reveal that VOCs of *Pseudomonas* sp. Ba35 strain could lead to some morphological abnormalities in *A. tumefaciens* cells. As well, GC–MS analysis indicated that *Pseudomonas* sp. Ba35 Specifically produce linalool and alpha-terpineol. Previous studies have shown that both of these compounds had strong antibacterial activity and induced the morphological change of bacteria^41–43^.

In conclusion, in the present study, it was shown that VOCs produced by endophytic bacteria could inhibit motility and virulence traits of *A. tumefaciens*, consequently causing some morphological abnormalities in *A. tumefaciens* cells, as well as reducing the attachment of cells to the roots of grape. The rhizosphere is a relatively closed environment favorable for a high volatile activity. The VOCs can spread to a long-distance and then produce an antibacterial environment. Therefore, such antibacterial volatile compounds may inhibit *A. tumefaciens* movement in the rhizosphere, also bring negative effects on attachment, and infection of bacterial cells via root tissues. Therefore, having information on the mechanisms of antibacterial activity of these compounds is necessary to understand the microbial interactions in natural environments.

## Methods

### Bacterial strains and plant materials

The endophytic bacteria *Pseudomonas* sp. Ou22 (GenBank Acc. No. MK114602), *Pantoea* sp. Sa14 (GenBank Acc. No. MK114617)*, Enterobacter* sp. Ou80 (GenBank Acc. No. MK114611), *Serratia* sp. Ba10 (GenBank Acc. No. MK114621), *Pseudomonas* sp. Ba35 (GenBank Acc. No. MK114598), and *Pseudomonas* sp. Sn48 (MK114596) isolated from the domesticated and wild-growing grapevine, as well as *Agrobacterium tumefaciens* Gh1 (GenBank Acc. No MZ647525), which exhibited virulence in grapevine were used in this study^21^. Accordingly, these bacteria were grown on nutrient agar (NA) medium and then stored at 4–6 °C as a working stock or grown in nutrient broth (NB) medium for 24 h at 26–28 °C with shaking. Finally, sterile glycerol was added to the final concentration of 20% and then stored at -20 °C for long-term storage.

Grapevine plantlets, cultivars Rashe, and Bidane sefid were kindly provided by the department of Horticultural science, University of Kurdistan, Iran. For the collection of plantlets, all relevant permissions have been obtained where applicable. The experimental research on grapevine plantlets conducted in this study complies with relevant institutional, local, and national regulations.

### Evaluation of the antibacterial activity of VOCs produced by endophytic bacteria

The antibacterial activity of VOCs produced by endophytic bacteria against *A. tumefaciens* Gh1 was assessed on nutrient agar medium using a dual-culture technique. The overnight growth of the endophytic bacteria (which was adjusted to the concentration of OD_600_ ≃ 1.0) was streaked on one side of the plate, while the opposite side of the plate was spot inoculated with 10 µl of the pathogen (OD_600_ ≃ 0.8). In the control, the pathogen was cultured alone. Thereafter, the plates were sealed with parafilm and then incubated at 26–28 °C for 7 days. The diameter of the *A. tumefaciens* Gh1 colonies was measured and the colony numbers per plate were calculated as well^20^. Three replications were performed for each treatment.

### Effect of VOCs on crown gall disease development

Grapevine plantlets were potted in pots containing steam-sterilized soil (consisting of 50% sand, 20% clay, 30% peat, pH 7.2). The suspension of *A. tumefaciens* cells with or without exposure to the VOCs of endophytic bacteria for three days at 26–28 °C was prepared in sterile water (density of OD_600_ ≃ 1.0). The stems were punctuated with a sterile toothpick and 20 µl was inoculated (between the third and fourth internodes) using a sterile syringe. Plantlets were incubated in a greenhouse (95% humidity, 25–26 °C, 16 h/8 h day/night photoperiod) and gall formation was recorded up to 30 days and the fresh gall weight was measured. Notably, each treatment was tested on three separate grapevine plantlets.

### Scanning electron microscopy (SEM)

Scanning electron microscopy (SEM) was used to observe external morphological changes of the *A. tumefaciens* Gh1 cells. Bacterial cells with or without exposure to the VOCs of endophytic bacteria for three days at 26–28 °C were collected into Eppendorf tubes, washed twice with 0.1 M phosphate-buffer Saline (PBS, pH:7.2), and centrifuged (10 min, 7000 rpm, 4 °C). Afterward, the cells were spread onto the clean slide. After fixation in 2.5% glutaraldehyde solution for 4 h at room temperature, the sample was washed three times with PBS, followed by two rinses with sterilized-distilled water. Serial dehydration was done in ethanol solutions of 30%, 50%, 70%, 80% 90%, and 100%, for 10 min, each time followed by 100% ethanol for 1 h. The samples were then conducted by the freeze-drying process at -40 °C for 3 h. Finally, the samples were coated with gold, and electron micrographs were taken using a TSCAN SEM system (TSCAN SEM, TSCAN, Czechoslovakia).

### Swarming, swimming, and twitching motility behaviors

The motility behaviors of the *A. tumefaciens* Gh1 cells exposed to VOCs produced by endophytic bacteria were tested using divided Petri plates. The overnight growth of the *A. tumefaciens* Gh1 was adjusted to an approximate concentration of OD_600_ ≃ 0.8, and then 2 µl was spotted onto one compartment of the divided plates containing NB medium plus agar (0.2-, 0.7-, 1.6%) for swimming, swarming, and twitching motility, respectively. In the other compartment, 30 µl of the endophytic bacteria with the approximate concentration of OD_600_ ≃ 1.0was streaked on NA medium. The plates were incubated at 26–28 °C and the halo diameters of swarming, swimming, and twitching motility were examined after 48 and 72 h. The experiment was done in three replications^20^.

### Biofilm formation assay

The biofilm formation ability of *A. tumefaciens* Gh1 cells exposed to the VOCs produced by endophytic bacteria was investigated in polypropylene tubes. For this purpose, 10 µl of a 24-h culture of endophytic bacterial strains (OD_600_ ≃ 1.0) were cultured onto one compartment of the divided plates containing NA culture medium. In the other compartment, a microtube containing 190 μl of LB liquid culture medium that was inoculated with 10 μl of *A. tumefaciens* Gh1 (OD_600_ ≃ 0.8) was placed vertically. The plates were then sealed with parafilm and kept at 26–28 °C for 24 h. Thereafter, 25 μl of 1% crystal violet solution was added to each microtube and then kept at room temperature for 15 min. The microtubes were then rinsed twice with sterile water. Subsequently, 2 × 200 μl of 95% ethanol was added to each tube, the resulting volume was brought to 1 ml with sterile-distilled water and the absorbance was measured at 540 nm using a spectrophotometer (SPECORD 210, Analytik Jena, Germany). *A. tumefaciens* Gh1 cells without any exposure to VOCs were used as a control. Accordingly, the experiment was performed in a completely randomized design with three replications^44^.

### Chemotaxis assay

For the chemotaxis assay, endophytic bacteria were streaked onto one compartment of the divided plates containing NA medium as described earlier. In the other plate compartment, chemotaxis buffer medium (0.1 mM EDTA, 10 mM K_2_HPO_4_, 0.35% agar, pH 7.2) was prepared, and 5 mm of the medium was also removed and then refilled with 50 µl of root extract of grapevine (cultivars Rashe, and Bidane sefid). Next, *A. tumefaciens* Gh1 cells were spot inoculated at a distance of 15 mm from the hole. The plates were sealed with parafilm and then incubated at 26–28 °C. The movement of the *A. tumefaciens* Gh1 cells towards the root extract was measured by colony diameters as well as counting the CFU/ml of the cell on NA. This experiment was performed in three replicates^45^.

### Grapevine root attachment assay

The attachment of *A. tumefaciens* Gh1 cells to grapevine roots was assessed after the exposure to VOCs of endophytic bacteria for 72 h. For this purpose, healthy and wounded grapevine roots were submerged in *A. tumefaciens* Gh1 cell suspensions (adjusted to about OD_600_ ≃ 0.8) with or without exposure to VOCs of endophytic bacteria for 1 h at 28 °C. Thereafter, 3 to 5 mm were separated from the root tips and then placed individually in 10 ml of sterile distilled water. After stirring for 5 s, the excess water was removed and the roots were placed in 500 μl of 10 mM HEPES with pH = 7 (N-2-hydroxyethylpiperazine-N'-2-ethane sulfonic acid). The obtained suspension was cultured onto NA medium and the CFU/ml was counted. Notably, three replications were considered for each treatment^30^.

### Identification of VOCs produced by endophytic bacteria using GC–MS analysis

In order to collect the VOCs produced by each endophytic bacteria, a three-compartment plate was used. One compartment, containing NA medium was streaked with 100 µl (OD_600_ ≃ 1.0) overnight growth of each endophytic bacteria, the second compartment containing NA medium was spot inoculated with 5 µl (OD_600_ ≃ 0.8) of *A. tumefaciens* Gh1, and the third compartment was filled with 0.3 g of sterile activated charcoal to adsorb the VOCs. As well, the same experimental design without endophytic bacteria or activated charcoal was used as a control. Subsequently, the plates were sealed with parafilm and then incubated at 25–26 °C for 72 h. The activated charcoal traps were transferred into glass vials and ethyl acetate (1: 1.25 W/V) was added to them. The adsorbed VOCs were extracted by shaking for 20 min, followed by the centrifugation (2500 g, 5 min) and the supernatants were analyzed by using gas chromatography device connected to a mass spectrometer (Agilent 7890B GC System / 5977A MSD).

Thereafter, one microliter of the sample was injected into HP-5 ms column (30 m × 0.25 mm, 0.25 Micron), the initial column temperature was set at 60 °C, which was then increased to 260 °C at a rate of 7 °C/min, and held for 5 min. The mass spectrometer was operated in the electron ionization mode at 70 eV, with continuous scanning from 50 to 550 m/z. As well, Helium carrier gas with a purity of 99.999%, a 34 psi pressure, and a flow rate of 1 ml/min was used at this stage. The compounds were identified by comparing their mass spectra with the databases of the device, including the National Institutes of Standards and Technology (NIST) and Wiley databases, along with comparing the inhibition indices and the failure pattern reported for them^46^.

#### Statistical analysis

To evaluate the significance of the performed treatments, the data from each experiment were analyzed by the using analysis of variance (ANOVA), followed by Least-Significant Difference (LSD) test (*P* = 0.05), using SAS ver. 9.1 statistical software. All the experiments were conducted in a completely randomized design in three replication. Graphs and figures were plotted using Excel program.

## Supplementary Information

Supplementary Information.

## Data Availability

All data that support the findings of this study are available from the corresponding author upon reasonable request.

## References

[1] Djellout H, Raio A, Boutoumi H, Krimi Z (2020). Bacillus and *Pseudomonas* spp. strains induce a response in phenolic profile and enhance biosynthesis of antioxidant enzymes in *Agrobacterium tumefaciens* infected tomato plants. Eur. J. Plant Pathol..

[2] Thompson MG (2020). *Agrobacterium tumefaciens*: A bacterium primed for synthetic biology. BioDesign Res..

[3] Kawaguchi A (2019). Biological control agent *Rhizobium* (=*Agrobacterium*) *vitis* strain ARK-1 suppresses expression of the essential and non-essential *vir* genes of tumorigenic *R*. *vitis*. BMC Res. Not..

[4] Heindl JE (2014). Mechanisms and regulation of surface interactions and biofilm formation in Agrobacterium. Front. Plant Sci..

[5] Burr TJ, Otten L (1999). Crown gall of grape: Biology and disease management. Ann. Rev. Phytopathol..

[6] Kawaguchi A, Inoue K, Tanina K, Nita M (2017). Biological control for grapevine crown gall using nonpathogenic *Rhizobium vitis* strain ARK-1. Proc. Japan Acad. Ser. B Phys. Biol. Sci..

[7] Xue QY (2009). Evaluation of the strains of *Acinetobacter* and *Enterobacter* as potential biocontrol agents against *Ralstonia* wilt of tomato. Biol. Control.

[8] Farrand SK, Baker RR, Dunn PE (1990). *Agrobacterium radiobacter* strain K84: A model control system. New Directions in Biological Control: Alternatives for Suppressing Agricultural Pests and Diseases.

[9] Chen F (2007). Biological control of grape crown gall by *Rahnella aquatilis* HX2. Plant Dis..

[10] Dandurishvili N (2011). Broad-range antagonistic rhizobacteria *Pseudomonas fluorescens* and *Serratia plymuthica* suppress *Agrobacterium* crown gall tumours on tomato plants. J. Appl. Microbiol..

[11] Yunus FN (2016). Antagonistic activity of *Pseudomonas fluorescens* against fungal plant pathogen *Aspergillus niger*. Sci. Lett..

[12] Compant S (2005). Use of plant growth-promoting bacteria for biocontrol of plant diseases: Principles, mechanisms of action, and future prospects. Appl. Environ. Microbiol..

[13] Garbeva P, Weisskopf L (2020). Airborne medicine: Bacterial volatiles and their influence on plant health. New Phytol..

[14] Zarraonaindia I (2015). The soil microbiome influences grapevine-associated microbiota. mBio.

[15] Schulz S, Dickschat JS (2007). Bacterial volatiles: The smell of small organisms. Nat. Prod. Rep..

[16] Rajer FU (2017). Volatile organic compounds produced by a soil-isolate, Bacillus subtilis FA26 induce adverse ultra-structural changes to the cells of *Clavibacter michiganensis* ssp. *sepedonicus*, the causal agent of bacterial ring rot of potato. Microbiology.

[17] Xie A (2018). Antibacterial effects of volatile produced by *Bacillus* strain D13 against *Xanthomonas oryzae* pv. *oryzae*. Mol. Plant Pathol..

[18] Raza W (2016). Volatile organic compounds produced by *Pseudomonas fluorescens* WR-1 restrict the growth and virulence traits of *Ralstonia solanacearum*. Microbiol. Res..

[19] Raza W (2016). Response of tomato wilt pathogen *Ralstonia solanacearum* to the volatile organic compounds produced by a biocontrol strain *Bacillus amyloliquefaciens SQR*-9. Sci. Rep..

[20] Tahir HA (2017). Bacillus volatiles adversely affect the physiology and ultra-structure of *Ralstonia solanacearum* and induce systemic resistance in tobacco against bacterial wilt. Sci. Rep..

[21] Asghari S (2019). Screening of endophytic bacteria isolated from domesticated and wild growing grapevines as potential biological control agents against crown gall disease. Biocontrol.

[22] Asghari S (2020). Induction of systemic resistance to *Agrobacterium tumefaciens* by endophytic bacteria in grapevine. Plant Pathol..

[23] Weisskopf L, Ryu CM, Raaijmakers J, Garbeva P (2016). Editorial: Smelly fumes - volatile-mediated communication between bacteria and other organisms. Front. Microbiol..

[24] Tahir HA (2017). Plant Growth Promotion by Volatile Organic Compounds Produced by *Bacillus subtilis* SYST2. Front. Microbiol..

[25] Gürtler H (1994). Albaflavenone, a sesquiterpene ketone with a zizaene skeleton produced by a streptomycete with a new rope morphology. J. Antibiot..

[26] Chowdhury FT, Islam MR, Islam MR, Khan H, Jha S (2019). Diversity of plant endophytic volatile organic compound (VOC) and their potential applications. Endophytes and Secondary Metabolites. Reference Series in Phytochemistry.

[27] Merritt PM, Danhorn T, Fuqua C (2007). Motility and chemotaxis in *Agrobacterium tumefaciens* surface attachment and biofilm formation. J. Bacteriol..

[28] Harighi B (2008). Role of CheY1 and CheY2 in the chemotaxis of *A. tumefaciens* toward acetosyringone. Curr. Microbiol..

[29] Harighi B (2009). Genetic evidence for CheB-and CheR-dependent chemotaxis system in *A. tumefaciens* toward acetosyringone. Microbiol. Res..

[30] Brisset MN, Rodriguez-Palenzuela P, Burr TJ, Collmer A (1991). Attachment, chemotaxis, and multiplication of *A. tumefaciens* biovar 1 and biovar 3 on grapevine and pea. Appl. Environ. Microbiol..

[31] Abarca-Grau AM, Penyalver R, López MM, Marco-Noales E (2010). Pathogenic and non-pathogenic *Agrobacterium tumefaciens*, *A. rhizogenes* and *A. vitis* strains form biofilms on abiotic as well as on root surfaces. Plant Pathol..

[32] Faridha Begum I, Mohankumar R, Jeevan M, Ramani K (2016). GC-MS analysis of bio-active molecules derived from *Paracoccus pantotrophus* FMR19 and the antimicrobial activity against bacterial pathogens and MDROs. Indian J. Microbiol..

[33] Ahsan T (2017). Extraction and identification of bioactive compounds (eicosane and dibutyl phthalate) produced by *Streptomyces* strain KX852460 for the biological control of *Rhizoctonia solani* AG-3 strain KX852461 to control target spot disease in tobacco leaf. AMB Express..

[34] Mohamad OAA (2018). Evaluation of the antimicrobial activity of endophytic bacterial populations from chinese traditional medicinal plant licorice and characterization of the bioactive secondary metabolites produced by *Bacillus atrophaeus* against *Verticillium dahliae*. Front. Microbiol..

[35] Létoffé S (2014). Aerial exposure to the bacterial volatile compound trimethylamine modifies antibiotic resistance of physically separated bacteria by raising culture medium pH. mBio.

[36] Singh VK, Kavita K, Prabhakaran R, Jha B (2013). Cis-9-octadecenoic acid from the rhizospheric bacterium *Stenotrophomonas maltophilia* BJ01 shows quorum quenching and anti-biofilm activities. Biofouling.

[37] Liu S (2008). Biological control of phytopathogenic fungi by fatty acids. Mycopathologia.

[38] Casillas-Vargas G (2021). Antibacterial fatty acids: An update of possible mechanisms of action and implications in the development of the next-generation of antibacterial agents. Prog. Lipid Res..

[39] Haas D, Defago G (2005). Biological control of soil-borne pathogens by fluorescent pseudomonads. Nat. Rev. Microbiol..

[40] Jishma P (2017). Strain-specific variation in plant growth promoting volatile organic compounds production by five different *Pseudomonas* spp. as confirmed by response of Vigna radiata seedlings. J. Appl. Microbiol..

[41] Li L (2015). Antibacterial activity of α-terpineol may induce morphostructural alterations in *E. coli*. Braz. J. Microbiol..

[42] Gao Z (2019). Anti-listeria activities of linalool and its mechanism revealed by comparative transcriptome analysis. Front. Microbiol..

[43] Liu X (2020). Antibacterial activity and mechanism of linalool against *Pseudomonas aeruginosa*. Microb. Pathog..

[44] O’Toole GA, Kolter R (1998). (1998) Initiation of biofilm formation in *Pseudomonas fluorescens* WCS365 proceeds via multiple, convergent signaling pathways: A genetic analysis. Mol. Microbiol..

[45] Sreeramanan S (2006). Chemotaxis movement and attachment of *Agrobacterium tumefaciens* to banana tissues. Biotechnology.

[46] Dewanjee S (2015). Bioautography and its scope in the field of natural product chemistry. J. Pharm. Anal..

